# Supplementary material for: Identification and Validation of eRNA as a Prognostic Indicator for Cervical Cancer
Source: Biology (Basel). 2024 Mar 29;13(4):227. doi: 10.3390/biology13040227 (PMC11048606; doi:10.3390/biology13040227)
Supplement: Supplementary file 1 [file biology-13-00227-s001.zip › biology-2884803-supplementary.pdf]

Supplementary Materials

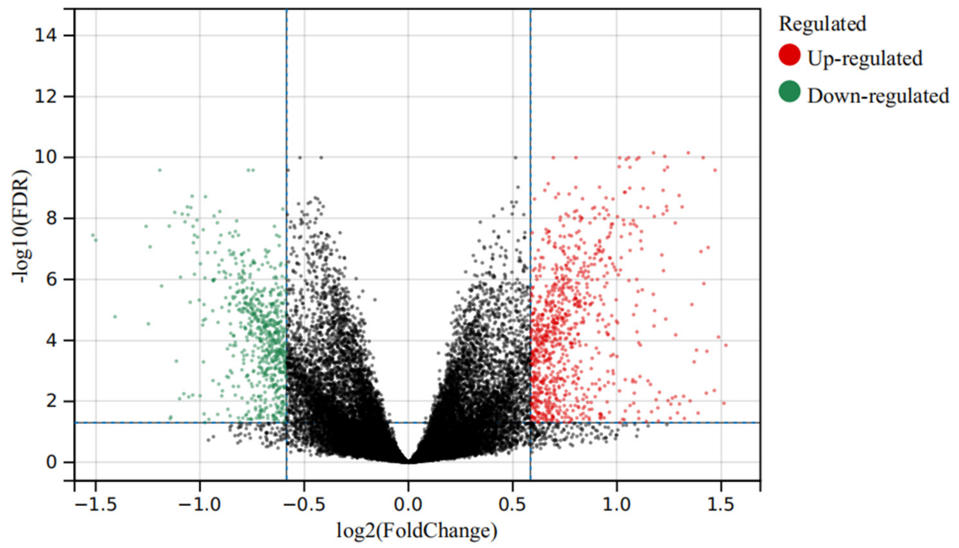

(**a**)

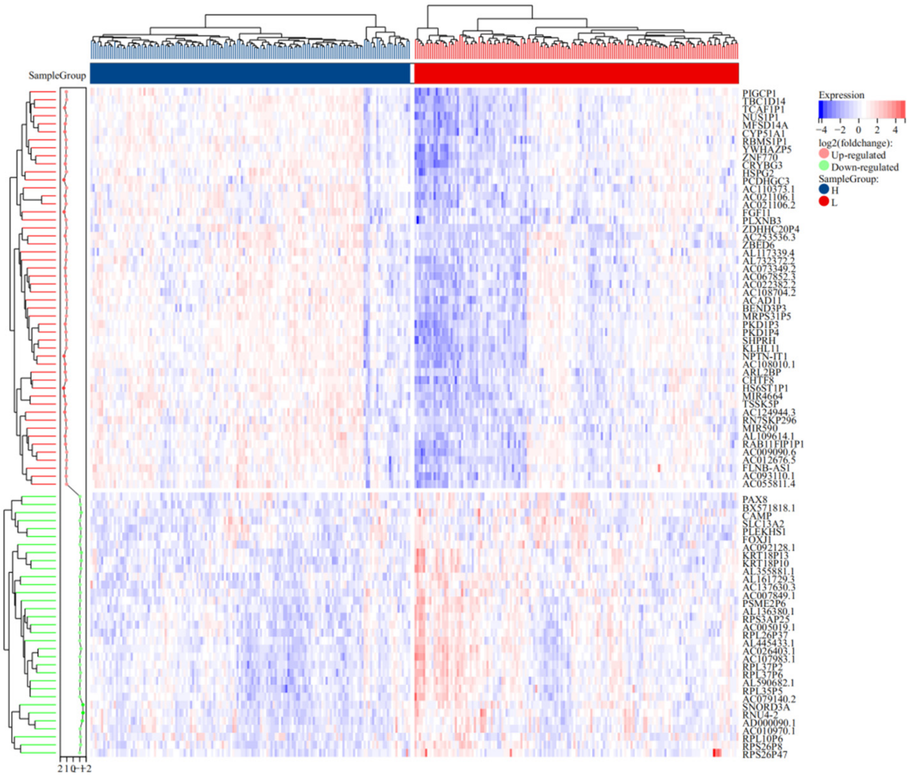

(**b**)

Figure S1. Analysis of gene differential expression in CESC high- and low-risk groups. (**a**) Volcano plot comparing differential gene expression analysis in high- and low-risk groups ($p < 0.05$ and $|\log2FC| > 2$). Red represents up-regulation and green represents down-regulation. (**b**) Heatmap of differential expression analysis of the first 50 genes in high- and low-risk groups. Red represents up-regulation and blue represents down-regulation.

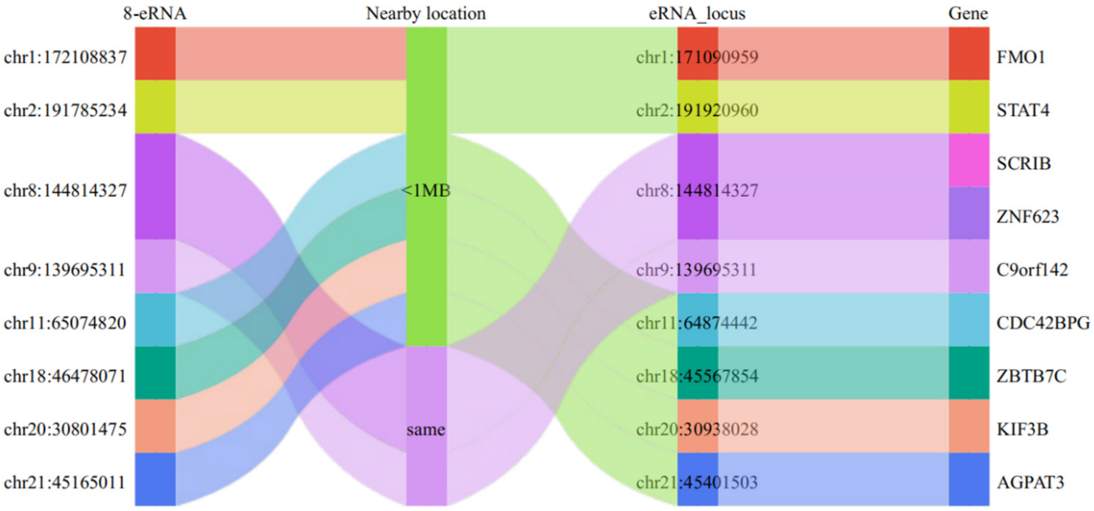

(**a**)

Figure S2. Refer to the eRNA corresponding gene obtained by the TCEA portal. (**a**) Sankey Diagram.

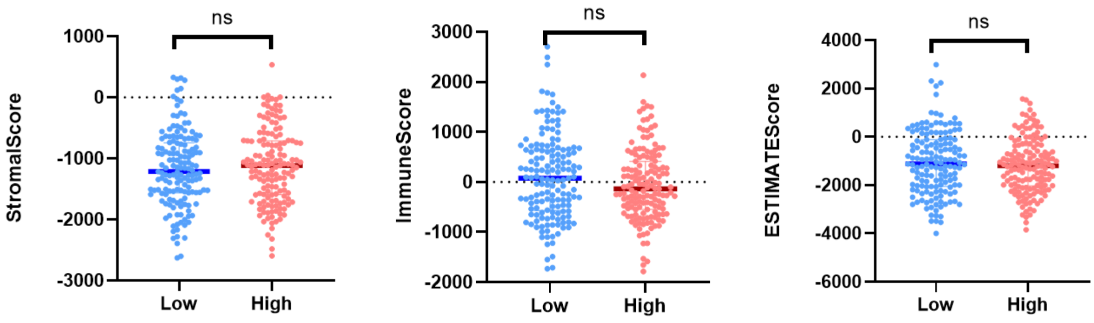

(**a**) (**b**) (**c**)

Figure S3. Analysis of tumor immune microenvironment between high- and low-risk groups. (**a**) The ESTIMATE analysis calculated stromal cell score, (**b**) immune cell score, and (**c**) ESTIMATE score between high- and low-risk groups.

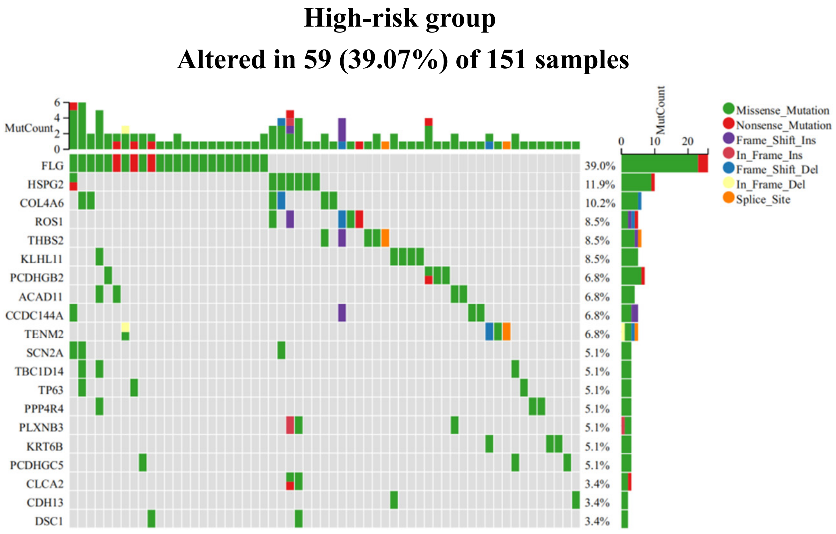

(**a**)

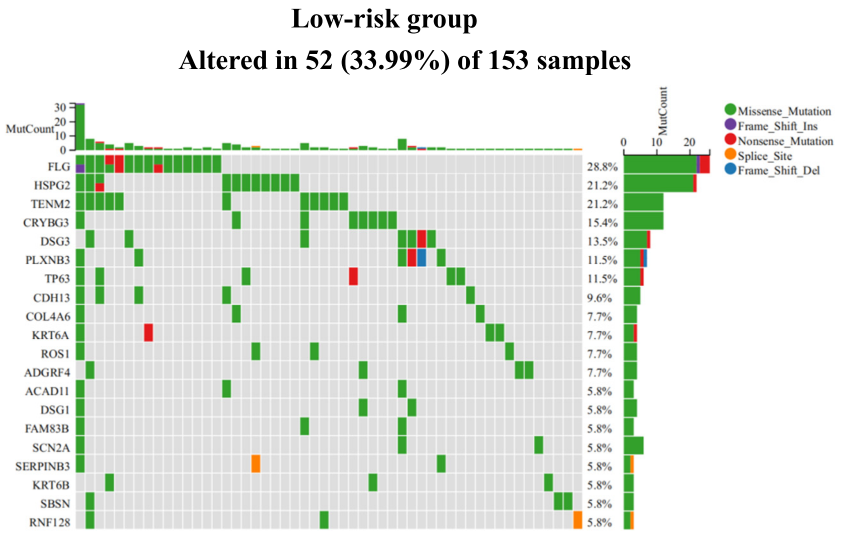

(b)

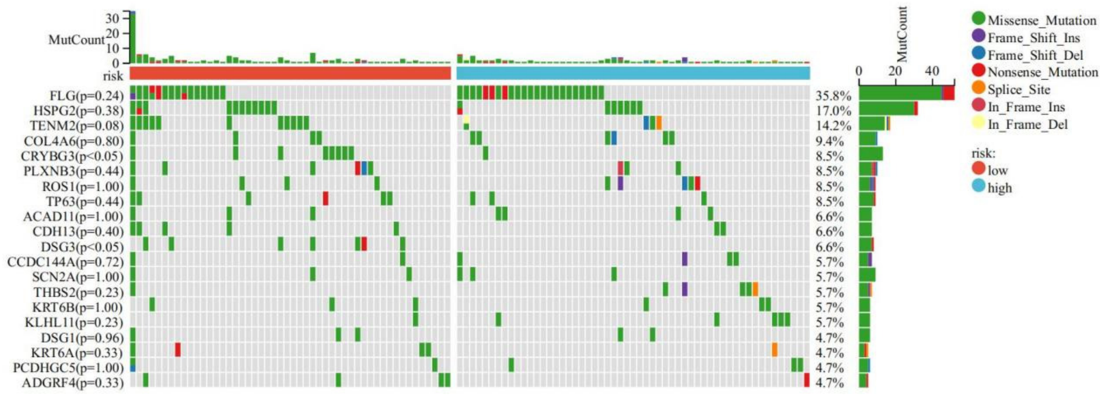

(c)

Figure S4. Analysis of genomic changes between high- and low-risk subgroups. Panels (**a**,**b**) show that only about 30% of the genes are mutated. (**c**) The chi-square test was used to evaluate the frequency of mutations in the first 20 genes in the two groups of samples; it was found that there seemed to be no significant difference in mutations between the two groups, and only the p-values of CRYBG3 and DSG3 genes were less than 0.05.
